# Digital Markets, Local Products: Psychological Drivers of Buying Nomadic Local Foods Online

**DOI:** 10.3390/foods14203468

**Published:** 2025-10-11

**Authors:** Samira Esfandyari Bayat, Armin Artang, Naser Valizadeh, Morteza Akbari, Masoud Bijani, Pouria Ataei, Imaneh Goli

**Affiliations:** 1 Department of Agricultural Extension and Education, College of Agriculture, Tarbiat Modares University (TMU), Tehran 141556619, Iran; s.esfandiyaribayat@modares.ac.ir (S.E.B.); mbijani@modares.ac.ir (M.B.); 2 Faculty of Entrepreneurship, University of Tehran, Tehran 1439813141, Iran; artangarmin1988@gmail.com (A.A.); mortezaakbari@ut.ac.ir (M.A.); 3 Department of Agricultural Extension and Education, School of Agriculture, Shiraz University, Shiraz 7134845794, Iran; 4 Department of Socio-Economic and Agricultural Extension Research, Fars Agricultural and Natural Resources Research and Education Center, Agricultural Research, Education & Extension Organization (AREEO), Shiraz 7155863511, Iran; p.ataei@areeo.ac.ir; 5 Department of Economics and Rural Development, Gembloux Agro-Bio Tech, University of Liège, 5030 Gembloux, Belgium; imaneh.goli@uliege.be

**Keywords:** online food shopping, local foods, Theory of Planned Behavior (TPB), hedonic and utilitarian values, consumer purchase intention

## Abstract

E-commerce is quickly increasing purchasing behavior across the globe, but little is known about how psychological paradigms underscore online buying intentions for locally essential items as nomadic local foods. The primary goal of this research is to examine the effects of some important psychological constructs and motivational values on predicting consumers’ intention to purchase nomadic and local foods via online e-commerce platforms, such as Ashayershop. This study followed the Theory of Planned Behavior (TPB) and looked at direct and mediated effects of attitudes, perceived behavioral control, and subjective norms on intention to purchase. Structural Equation Modeling (SEM) was conducted, based on data collected from a representative sample of consumers who were familiar with online shopping for local foods. The results highlight that attitude towards online shopping for local foods was the strongest direct predictor of intention to purchase (β = 0.383, T = 9.487, *p* < 0.001). Perceived behavioral control (β = 0.220, T = 5.316, *p* < 0.001), hedonic value (β = 0.213, T = 4.907, *p* < 0.001), utilitarian value (β = 0.187, T = 3.719, *p* < 0.001), and subjective norms (β = 0.149, T = 3.493, *p* < 0.001), received a significant positive effect on intention. In addition, hedonic and utilitarian values bountifully mediated the relation between psychological antecedents (attitudes, perceived behavioral control, and subjective norms) and purchase intention. For instance, attitude indirect effect via hedonic value was β = 0.080 (T = 3.783, *p* < 0.01), and indirect effect via utilitarian value was β = 0.040 (T = 3.058, *p* < 0.01), indicating the importance of these values as mediators. This research makes a contribution to the literature by showing that motivational values serve as not only an outcome but also as cognitive–affective mediators in the behavioral process thus expanding the TPB in the context of digital food markets. In general, these results provide valuable insights to e-commerce platforms and policymakers who desire to promote consumer engagement with products stemming from culture and tradition on line by developing new integrated strategies that address the cognitive, emotional, and social components.

## 1. Introduction

Internet statistics have reported that in 2022, there were more than five billion internet users. Comparing this number with internet users in 2000 shows a significant growth in internet usage [1]. The expansion and advancement of internet technologies have not only had a profound impact on people’s lives but have also greatly affected businesses, particularly online businesses [2,3,4]. In other words, it can be claimed that online commerce is one of the main driving forces of global growth [5,6,7]. Important factors such as changes in consumer lifestyles, technological advancements, increased wealth and consumer knowledge, and rapid financial growth around the world have contributed to the growing popularity of online commerce [5,8,9]. As the internet has become an essential tool for communication and commerce worldwide, online shopping is considered a suitable priority for consumers [10,11,12]. Online shopping can be regarded as the third most popular activity among internet users after email and web browsing [10,13]. The number of online shoppers is rapidly increasing, and accordingly, the use of online shopping platforms is also on the rise [14,15,16].

One of the most important prerequisites for launching any business is considering online buying and selling as a strategy to increase revenue and enhance competitiveness [17,18]. Eectronic commerce and businesses, educating consumers and online buyers is an undeniable prerequisite for development [19]. Online shopping allows customers to purchase goods and services from any location (even in rural areas) and at any time. This shopping method also helps consumers reduce the time and effort required for shopping [20,21]. The time and location flexibility of online shopping leads to avoiding crowds, reducing waiting times, and saving the energy needed for shopping, as consumers do not physically go to stores and shopping centers [22,23]. Moreover, this type of shopping also has psychological benefits, as consumers enjoy the advantages of accessing products, brands, and stores not available in their place of residence or work [24,25]. The online commerce market is currently expanding, and buyers are trying to order their required items, including food products, online [26,27]. Online buying and selling applications are considered key tools in the development of businesses related to food product trade [28,29].

Unlike traditional advertising, e-commerce of food products represents an innovative model of marketing conducted through online buying and selling applications and platforms [30]. Social support for these platforms links e-commerce to the sale of farmer-assisting agricultural/local products [31,32,33,34]. For example, during the COVID-19 pandemic, e-commerce platforms significantly helped farmers sell food products through marketing activities [35,36,37]. In other words, the effective use of these platforms helped reduce the issue of accumulation and unsold agricultural food products in certain regions [38]. As a result, the use of these platforms mitigated the pandemic’s impact on farmers and reduced their income losses. Around the world, various applications have been designed for consumers so they can easily purchase food products without spending much time and energy [39,40]. Given the development of internet infrastructure in many countries, sellers and online businesses are trying to increase customer usage of these apps [41,42]. To that end, identifying and investing in the factors that contribute to increased consumer intention to use online shopping platforms and apps for food products is of great importance to them [43,44,45].

Iran is also one of the developing countries where the use of online food buying and selling platforms holds significant importance [46,47]. For instance, in recent years, numerous applications and platforms such as Snapfood, Changal and Chelivery, and others have been developed for food commerce and have gained public acceptance [48]. However, it is noteworthy that most of these online shopping platforms generally offer food products produced and marketed by well-known companies [49]. In other words, rural and nomadic populations, who typically produce local and natural food products, are often unable to use these platforms to sell their products [50]. However, since 2020, an online platform called Ashayershop has been launched by the Iranian Nomadic Affairs Organization, which is specifically focused on the online sale of nomadic food products and even handicrafts. This system has enabled nomads to avoid physically attending local markets to sell their products. The platform functions in a way that allows nomads to access it, receive a code, and continuously upload samples of their products and goods. During the COVID-19 pandemic, this food e-commerce platform helped nomads market a large portion of their food products without having to travel to marketplaces, without incurring costs, and at suitable times [51]. Although the use of this platform among local food producers and consumers is rapidly increasing, it still has not achieved widespread popularity among consumers compared to other online platforms. One possible reason may be the lack of sufficient understanding of the predictors of consumer intention to purchase local food products from this platform. More surprisingly, no study has been conducted in Iran to identify and analyze the factors determining consumer intention to buy food online through the Ashayershop platform. Given the importance of this issue, the present research study aims to examine the determinants of consumer intention to purchase food online through the Ashayershop platform in Iran.

This study is innovative and significant from several perspectives. First, it is thematically novel, as no prior research in Iran has specifically examined the determinants of consumers’ intention to purchase food products online through the Ashayershop platform. Second, the findings offer valuable insights into the motivational factors driving consumers to buy products produced by nomadic communities, which can, in turn, contribute meaningfully to the economic empowerment of nomads. Supporting the nomadic economy in Iran is particularly important, as these communities play a critical role in the production of essential food items such as milk, meat, honey, and their derivatives—including yogurt, cheese, and other dairy products. Identifying the determinants of consumer intention to purchase such foods via Ashayershop could enhance the sustainability of nomadic economies and thus strengthen food security in the country. Third, this research provides a causal mechanism linking psychological and behavioral variables to online purchase intention, offering a framework for designing more practical and effective interventions aimed at increasing consumer engagement with platforms like Ashayershop. In other words, the results of this study can assist consumer behavior change agents, the nomadic community, food policymakers, officials responsible for nomadic affairs in Iran, and economic planners in identifying the key variables that shape consumer purchase intentions. These findings also help inform the selection and sequencing of strategies to foster greater adoption of online food shopping through Ashayershop. Fourth, by investigating the psychological and social factors influencing online food shopping behavior, this study contributes to a deeper understanding of consumer preferences and values. As such, it can provide useful insights for nomadic producers and agricultural extension specialists in tailoring food production to better align with consumer tastes and expectations.

## 2. Theoretical Framework and Hypothesis Development

### 2.1. Theory of Planned Behavior

Various theories have been used to examine consumer intentions toward purchasing different products, among which one of the most well-known is the Theory of Planned Behavior (TPB) [52,53,54]. This theory was first introduced by Icek Ajzen in 1991 and is widely recognized as a key framework for understanding consumer motivation in the domain of food marketing [55,56,57]. TPB and its extended forms have been used by various researchers to investigate consumers’ online purchasing behaviors [58,59,60]. It is worth noting that before introducing TPB, Ajzen and Fishbein [61] developed the Theory of Reasoned Action (TRA) to describe individuals’ thinking and predict their intentions and future behavior [62,63]. Attitude and subjective norms are the two core constructs of TRA [64,65]. Later, Ajzen refined and extended this model in 1988 to address its limitations, and the new theory, TPB, was formally introduced to the academic and global community in 1991 [66,67,68,69,70]. While TRA explains entirely voluntary behaviors, TPB accounts for behaviors over which individuals may not have complete volitional control [62,63]. In other words, unlike TRA, TPB acknowledges that not all behaviors—such as consumer actions—are fully voluntary and under personal control. For example, an individual may have a strong intention to buy food online but may refrain from doing so under specific circumstances. Therefore, actual or perceived behavioral control can influence intention or moderate the relationship between intention and behavior [67]. According to TPB, constructs such as attitude, subjective norms, and perceived behavioral control can directly influence individuals’ intentions (future behavior) [71]. The popularity of TPB is largely attributed to its remarkable ability to explain individual behavioral intentions through a parsimonious framework consisting of attitudes, norms, and control structures [52,72].

In this theory, the intention to purchase a product using online platforms refers to an individual’s tendency to perform a specific action, or more specifically, the decision to buy a product or service through various online platforms [73,74]. This variable essentially reflects the possibility of purchasing a product based on personal evaluation and external factors [73,75]. Purchase intention is a key factor in predicting consumers’ actual behavior [73]. In fact, the intention to perform a specific behavior has a motivational impact on actual behavior and serves as its immediate precursor [52]. According to TPB, the stronger the intention to perform a behavior, the more likely it is that the behavior will be carried out. It is worth noting that consumers’ purchase intention is shaped through a cognitive process involving products or services. Therefore, studies related to consumers’ purchase intention primarily analyze antecedent variables [76].

### 2.2. Development of Hypotheses

#### 2.2.1. Attitude and Purchase Intention

Personal attitudes toward a behavior (such as online food purchasing behavior) refer to the degree of favorable or unfavorable evaluation of that behavior from the individual’s perspective [71,77,78,79,80]. This definition clearly highlights the emotional nature of attitude. According to TPB, behavioral beliefs—referring to an individual’s internal beliefs about the consequences of online purchasing behavior—can influence the formation of attitudes toward the actual behavior [2,69,81,82]. These beliefs vary from person to person depending on factors such as prior personal experiences, personality traits, and the context in which they live [2,83]. More specifically, numerous studies have supported the positive relationship between consumers’ attitudes toward purchasing food and their preferences and intentions [52,84,85]. For example, Sun [86] found that consumers’ attitudes toward healthy eating were strongly influenced by their health concerns. Attitudes toward online food purchasing via online platforms reflect customers’ thoughts and beliefs about the good (benefits) or bad (losses) outcomes of purchasing through online food delivery platforms [85,87]. In this way, consumers estimate the value of online food services before using them, so their attitudes toward the outcomes of using these platforms and their online shopping experience are very important [88]. Accordingly, this study hypothesized the following:

**Hypothesis** **1.** *Attitude toward online food purchasing from the Ashayershop platform has a positive and significant effect on the intention to purchase food online from the Ashayershop platform.*

#### 2.2.2. Subjective Norms and Purchase Intention

Subjective norms describe the perceived social pressure to perform a particular action [71,78,79,83]. Subjective norms regarding healthy food purchasing can significantly help predict the intention to engage in healthy eating behaviors [8,52,69,88]. According to the assumptions of the TPB theory, when consumers make purchases, they create a self-image that others interpret [71,89]. Therefore, they confront the image they want others to have of them [71,80,82,90]. Accordingly, this study hypothesized that:

**Hypothesis** **2.** *Subjective norms regarding online food purchasing from the Ashayershop platform have a positive and significant effect on the intention to purchase food online from the Ashayershop platform.*

#### 2.2.3. Perceived Behavioral Control and Purchase Intention

Perceived behavioral control refers to the perceived ease or difficulty of performing a behavior such as online food purchasing [71,77,83,85,87]. Perceived behavioral control is a variable added in TPB and represents the extent to which an individual perceives control over performing the target behavior [69,78,81,82,89,91]. Accordingly, this study hypothesized that:

**Hypothesis** **3.** *Perceived behavioral control regarding online food purchasing from the Ashayershop platform has a positive and significant effect on the intention to purchase food online from the Ashayershop platform.*

#### 2.2.4. Hedonic and Utilitarian Values with Online Purchase Intention

In addition to the three core variables of the TPB mentioned above, researchers have recently introduced many other variables into this theory to enhance its explanatory power in analyzing consumer purchasing behaviors. In other words, consumer intentions and behaviors are generally based on values, which fundamentally act as intrinsic and personal motives for purchasing a product [92]. Consumer values or orientations toward using online food purchasing platforms are constructs that have received significant attention from researchers [93]. Value can encompass broad economic and psychological components. However, some researchers define it based on the “price” and “quality” of the purchased product. Limiting the definition of value to price and quality may lead to errors in its measurement [94,95,96]. Some researchers (see [88,93]) argue that food consumers when purchasing food via online platforms may exhibit two types of value: hedonic value and utilitarian value. Babin and colleagues [97] were the first to introduce this concept [92].

Generally, utilitarian value refers to the consumer’s overall evaluation of the functional benefits of a product, while hedonic value is defined as the overall evaluation of the experiential benefits. Products that mainly have aesthetic and symbolic features may significantly influence hedonic choices, whereas the product’s functionality has a significant impact on utilitarian choices [98]. Hedonic value stems from the pleasure experienced during the purchasing process, while utilitarian value arises from obtaining the desired product during the purchasing process [99,100]. The impact of these values on consumers’ online purchase intentions have been supported by numerous studies [94,101,102,103,104,105,106]. It should be noted that online food purchasing through delivery platforms can provide both values to consumers [71]. However, some individuals may prioritize either hedonic or utilitarian value more significantly [107].

**Figure 1:**
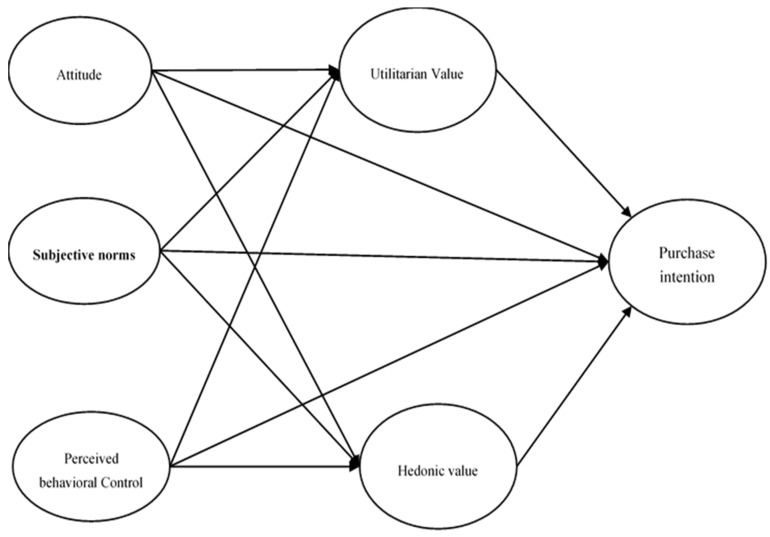
Research Model.

Utilitarian value refers to the consumer’s overall evaluation of the functional advantages and disadvantages of a product or service, such as food items. Consumers’ utilitarian values have been described as functional, task-oriented, and based on rational logic [99]. In other words, utilitarian values tend to dominate in planned purchases [107]. This value applies to consumers who believe that an online platform can improve the efficiency of their online shopping experience [73,108,109]. Utilitarianism refers to how a system or platform can enhance the performance of a specific process, such as online purchase behavior. The perceived functional value by customers is based on the practicality and evaluation of the service, which mainly depends on intrinsic features such as taste, color, and texture, as well as extrinsic features including price, brand, and the level of product or service advertising. The combination of these factors can influence customers’ purchase intentions through the construct of utilitarian value [76,110]. Consumers with utilitarian values seek the most efficient ways to shop [99]. Rintamaki et al. [111] argue that saving money and convenience are important components of utilitarian value for consumers. In other words, when consumers find a product on sale, perceive the price to be lower than competitors, or can complete their purchase effectively, their utilitarian value is strengthened [99]. Consumers’ perception of fair or unfair pricing significantly affects the strengthening or weakening of utilitarian value and their purchase intention [73]. Chen et al. [88] state that both utilitarian and hedonic values can act as direct predictors of online food purchase intentions from online platforms within the TPB framework. Accordingly, in this study, these two variables were added as new constructs to TPB that can directly influence consumers’ intention to purchase food online from the Ashayershop platform, and it was hypothesized that (Figure 1):

**Hypothesis** **4.** *Utilitarian value in the context of online food purchasing from the Ashayershop platform has a positive and significant effect on consumers’ intention to purchase food online from the Ashayershop platform.*

**Hypothesis** **5.** *Hedonic value in the context of online food purchasing from the Ashayershop platform has a positive and significant effect on consumers’ intention to purchase food online from the Ashayershop platform.*

#### 2.2.5. Mediating Effects of Hedonic and Utilitarian Values

Chen et al. [88] argue that utilitarian and hedonic values can mediate the relationship between the variables of attitude, subjective norm, and perceived behavioral control regarding online food purchasing from online platforms, and the intention to purchase food online. Therefore, it can be claimed that the three variables—attitude, subjective norm, and perceived behavioral control—have positive and significant effects on both utilitarian and hedonic values [112]. Since attitude reflects an individual’s beliefs toward a technology such as online shopping platforms, if consumers hold a positive attitude toward purchasing food through an online platform, it can be expected that they will perceive utilitarian and hedonic values in online shopping. Furthermore, social pressures or subjective norms of consumers can also influence the formation of values based on hedonic or utilitarian orientations. In other words, when consumers’ subjective norms generally emphasize the enjoyable experience of online food shopping through online platforms, it is expected that hedonic values will be strengthened. Conversely, if social norms focus on the benefits of utilitarian aspects of purchasing food via online platforms, the likelihood of strengthening utilitarian values increases. Moreover, perceived behavioral control—or the degree of difficulty or ease in purchasing food via online platforms—can affect consumers’ beliefs regarding the pleasure and functional utility of using these platforms. For example, when consumers perceive purchasing food through a platform as difficult, the hedonic and utilitarian values associated with that platform may decrease. Conversely, if consumers perceive the purchasing process as easy, it is expected that both hedonic and utilitarian values will be enhanced. Based on this, the present study hypothesizes that:

**Hypothesis** **6.** *Utilitarian value positively and significantly mediates the effect of attitude toward online purchasing on the intention to purchase online.*

**Hypothesis** **7.** *Utilitarian value positively and significantly mediates the effect of subjective norm toward online purchasing on the intention to purchase online.*

**Hypothesis** **8.** *Utilitarian value positively and significantly mediates the effect of perceived behavioral control toward online purchasing on the intention to purchase online.*

**Hypothesis** **9.** *Hedonic value positively and significantly mediates the effect of attitude toward online purchasing on the intention to purchase online.*

**Hypothesis** **10.** *Hedonic value positively and significantly mediates the effect of subjective norm toward online purchasing on the intention to purchase online.*

**Hypothesis** **11.** *Hedonic value positively and significantly mediates the effect of perceived behavioral control toward online purchasing on the intention to purchase online.*

## 3. Materials and Methods

### 3.1. Study Context

This study was conducted in Iran. The role of online buying and selling in Iran is also growing. In recent years, with the increase in internet users and the advancement of communication technologies, e-commerce in Iran has entered a new phase. The ability to buy and sell various goods and services online, the speed and convenience of transactions, easy access to information and price comparisons, secure payments, and after-sales services are some of the advantages that e-commerce offers to customers and businesses in Iran. Among these, the position of this business model within Iran’s nomadic communities is particularly important because they generally have relatively limited access to direct sales markets for their products. Meanwhile, demand for products from Iran’s nomadic communities is rapidly increasing. One reason for this is that products from nomadic societies are generally healthier. The approach of online commerce and buying and selling their products via apps has created a new opportunity for the economic development of these communities. Moreover, consumers seek the easiest ways to purchase the food they need. Therefore, this study aimed to investigate and analyze the determinants of consumers’ intention to buy local food online through one of the dedicated platforms for buying and selling nomadic local food products in Iran.

### 3.2. Population, Sampling, and Data Collection

The statistical population of this study consisted of 900 consumers who purchased local food products produced by nomads through the Ashayershop online platform during the first quarter of 2024. These consumers made their purchases using this platform and its features, which provide various advantages, characteristics, and information about the products. The purchased items included cheese, yogurt, honey, kashk (fermented whey), animal oil, medicinal herbs, herbal distillates, and more. While several of these products, like milk and cheese, can be purchased in supermarkets, their online purchase from Ashayershop represents additional values such as trust in the authenticity of the nomadic production, higher quality, support of local producers, and cultural significance associated with these items. In this sense, the geographical proximity of consumers to the original producers is not the sole determinant; rather, the perceived value and symbolic meaning of buying directly from nomads motivates online purchases.

Data were collected using a questionnaire designed by the authors themselves (a researcher-developed instrument). Before the main survey began, several important steps were taken to enhance the validity and accuracy of the data collection tool. First, the draft questionnaire was reviewed by a panel of experts in business psychology, marketing, consumer behavior, and e-commerce. This step was conducted by the first, second, and third authors, and the experts assessed the questionnaire’s face and content validity. As a result, items that were not relevant to measuring the study variables were removed, and some questions were revised to improve clarity and ease of understanding for respondents. In the second step, necessary coordination was made with the managers and officials of the Ashayershop platform as well as representatives from the sales and customer service departments to allow the authors to conduct preliminary assessments. Additionally, conversations were held with some platform users to gather their perspectives on potential challenges and obstacles in online food purchasing. Throughout these conversations, users indicated that freshness, the convenience of home delivery when time is short, and the cultural uniqueness of nomadic food products were some of the primary reasons for ordering online, separating these products from typical supermarket alternatives. Third, the questionnaire was piloted among a selected group of platform users to identify the time required to complete it, understandability of the questions, and any possible barriers in the response process, allowing for final adjustments. Finally, in the fourth step, the target population of active Ashayershop users was identified, and a suitable sample size of 269 was determined using the Krejcie and Morgan [113] table. This means that from the total population of 900 consumers during the first quarter of 2024, a scientifically determined sample of 269 individuals was drawn. The reason for not contacting all 900 consumers is that in social science research, full enumeration is rarely feasible due to cost, time, and resource limitations; therefore, a representative sample is selected to ensure both practicality and methodological rigor. The sample size of 269 was not arbitrary but corresponds exactly to the value recommended by the Krejcie and Morgan table for a population of 900, ensuring statistical sufficiency and representativeness.

Sampling was carried out using a systematic random sampling method, where individuals were selected from an unranked list at regular intervals and in a specific order. This method not only ensures a more even distribution of the sample across the population but also provides greater accuracy compared to simple random sampling. The phrase “more even distribution across the population” refers to the systematic spreading of selected respondents across the entire consumer list rather than relying on random clustering, ensuring that the sample captures the diversity of the population in terms of demographics and purchase behavior. This was measured by maintaining consistent selection intervals across the list, reducing the likelihood that certain subgroups (e.g., highly active users) would be overrepresented. Even if the data collection was collected online, the risk of interviewer error is inherently low in this method, but systematic random sampling was still undertaken to mitigate other biases as much as possible, such as the risk of bias towards highly active users or the risk of clustering with respondents that exhibited the same purchasing attributes; by spreading selection evenly across the full consumer list, systematic random sampling allowed for a high representativeness and limited the risks of unintentional bias and improved the dependability of the resulting data. Structurally and operationally, it is simpler to implement, reduces the likelihood of interviewer error, and saves costs, making it especially suitable for field studies with budget constraints. Most importantly, the 269 sampled respondents completed the questionnaire in full, meaning a 100% response rate. Additionally, respondents participated voluntarily and could have withdrawn at any time. Because all the participants completed the survey completely and voluntarily, the risk of non-response bias is omitted, and therefore so is the risk of any bias, validity or reliability with the data for analysis purpose. However, it’s important to keep in mind that detailed demographic structures such as age, gender, or lifestyle patterns of respondents were not systematically recorded in this phase, which limits the total analytic depth of consumer segmentation.

However, to guard against selection bias due to limiting the sample to Ashayershop consumers, there are a number of safeguards detailed below. First, systematic random sampling provided assurance that each of the consumers in the target population had the same probability of being selected to reduce the likelihood of certain sub-groups of consumers being over represented. Second, a piloting and review process of the questionnaire was conducted with the goal of clarity and to mitigate nonresponse bias. Third, while sampling, demographic characteristics and usage behaviors of consumers were monitored to limit the amount of homogeneity amongst the sample. Even though the study essentially focused upon Ashayershop users, the above process mitigated selection bias and provided a representative sample of the Ashayershop consumer base, which would ultimately increase the reliability and generalizability of the study findings.

### 3.3. Sample Demographics

In order to clarify the study sample, demographic data was also collected and is presented. In terms of age, slightly more than one third (34%) of the respondents were 25–34 years; a little slower was 28% were aged 35–44 years of age; followed by 22% aged 18–24 years, and lastly 16% were 45 years or older. The gender makeup of the respondents was close to equal at 52% female and 48% male. The results of the education level show the majority of respondents reported 61% at least had a Bachelor’s degree; while the remaining were 27% had a postgraduate degree and 12% did not continue or complete secondary or vocational studies. Although there was variation in income levels among respondents: 29% had corresponding monthly household income below the national median, 41% had monthly household incomes identified as belonging to the middle-income range, while 30% had monthly household incomes lying within the high-income brackets, demonstrating a full-range of purchasing power. Based on the descriptive data of the sample, 68% of respondents were situated in urban locales while 32% were distributed semi-urban/rural spaces, indicating both concentrated and dispersed participation within the digital marketplace. Also, 46% of respondents used Ashayershop 3–5 times per week, with 31% reporting that they utilized the platform once per week, while the remaining 23% reported occasional use (once or twice a month). This demographic information helps situate the findings and highlights how they were representative for the study of consumer behavior in digital cultural markets.

### 3.4. Measures

Table 1 presents the instruments used to measure the main constructs of the study. In this research, six key constructs of the conceptual framework were examined: attitude, subjective norms, perceived behavioral control, hedonic value, utilitarian value, and purchase intention. For each construct, between three to five items were developed, most of which were adapted from previous studies such as Ryu et al. [114] and Hanzaee and Rezaeyeh [115], or were self-developed and localized to fit the specific context of the research. For items adapted from previous studies, a translation and back-translation approach was utilized. A bilingual expert first translated the items into Persian, and then another expert independently back-translated into the original language. The experts discussed and settled any inconsistencies in the translated questionnaires. For items that were self-developed or significantly adapted, back-translation was not necessary; the items were developed in Persian and were culturally relevant and contextually specific, content validity was assured. The “attitude” construct includes three items that assess the consumer’s positive perception of purchasing from the Ashayershop platform. Subjective norms, measured by three items, evaluate the influence of social pressure and others’ expectations. Perceived behavioral control is assessed with three items focusing on the ease or difficulty of purchasing from the platform from the consumer’s perspective. Hedonic value comprises three items related to pleasure, excitement, and entertainment during the buying process, while utilitarian value includes three items addressing practical, economic, and health-related aspects of the products. Finally, the dependent variable, purchase intention, is measured with five items that refer to repurchasing intentions, recommending to others, and actively seeking purchase opportunities. This item design is comprehensive in terms of content and structure and aligns well with the theoretical literature on consumer behavior in the online environment.

With items adopted from existing studies, the decisions whether to keep, change or eliminate certain items were guided by alignment with theoretical literature and contextual relevance. Items that spoke directly to the construct of interest in the original scales and captured a culturally relevant meaning were kept. Items that were unclear, contained redundant wording, or were not relevant to the online purchase of local nomadic products were amended for clarity or discarded. There were also several new items added that captured contextual aspects, such as the historical and community aspects of the products. This ensured that both the measures being used were valid and comprehensive in the context of study. These steps, therefore, ensured that the final instrument maintained fidelity to the theoretical constructs while being fully relevant to Persian-speaking consumers.

To minimize the potential for social desirability bias, a number of measures were taken into consideration in designing the questionnaire and administering the survey. In the first instance, the survey was carried out totally online and anonymously to preserve an aspect of respondents’ anonymity and to ensure that their responses to survey prompts could not be traced or associated with their identities. In the second instance, the researcher provided clear directives indicating that there were neither ‘right’ nor ‘wrong’ answers, and told respondents to answer in an honest manner as a matter of fact, concerning their experiences. In the third instance, the researcher checked to see that the items were worded to avoid suggestions or judgment that could pressure someone to answer in a socially favorable manner. In the fourth instance, the researcher did provide voluntary participation in the study with the indication that participants could quit the study at any time. These measures further reduced the potential of respondents providing ‘socially desirable’ answers, which was particularly important for researchers studying authentic community and culturally relevant products.

### 3.5. Reliability and Validity of the Measures

In this study, the reliability and validity of the measurement scales were thoroughly examined to ensure that the instruments accurately and consistently captured the intended constructs. Internal consistency reliability was assessed using Cronbach’s alpha coefficients, which indicate the homogeneity and coherence of the items within each scale. Additionally, composite reliability was calculated to confirm that each construct demonstrated sufficient overall internal consistency. To establish construct validity, both convergent and discriminant validity were evaluated. Convergent validity was assessed through the Average Variance Extracted (AVE) index, which measures the extent to which items related to the same construct share variance. Discriminant validity was evaluated using the Fornell-Larcker criterion and HTMT to ensure that each construct was distinct and adequately differentiated from the others. The results of these assessments, detailed comprehensively in the findings section, confirm the soundness and reliability of the measurement tools used in this research.

### 3.6. Data Analysis

The collected data were analyzed using Structural Equation Modeling (SEM) based on the Partial Least Squares (PLS) approach. Several reasons justified the selection of this analytical method. First, the primary objective of the study was to predict consumers’ intentions to purchase local food products online, which necessitated using a technique capable of maximizing the explained variance by latent variables. PLS-SEM is considered one of the most effective methods for achieving this goal. Second, according to Hair et al. [116], PLS-SEM is highly suitable for analyzing complex or extended theoretical models. Third, the user-friendly nature of PLS software makes it a popular choice among researchers for its ease of implementation and interpretation. The analysis process involved two key stages. The first stage focused on assessing the measurement model, aiming to evaluate how well each indicator (item) explained its corresponding latent construct, ensuring the validity and reliability of the measurement instruments. The second stage involved assessing the structural model, where the relationships between latent variables were tested. In this phase, the model’s predictive ability regarding the dependent variable, i.e., purchase intention, was examined and validated. These two stages of analysis, along with the reported model evaluation indices, provided a rigorous framework for data analysis and supported the credibility of the study’s findings.

## 4. Results

In the present analysis, the assessment of internal consistency reliability and convergent validity was undertaken using four primary measures; namely, Cronbach’s Alpha, Composite Reliability (CR), Average Variance Extracted (AVE), and rho_A. Prior literature indicates that values greater than 0.70 are acceptable for Cronbach’s Alpha and CR indicating adequate levels of internal consistency, and values greater than 0.50 for AVE indicate acceptable levels of convergent validity [117,118]. The majority of constructs in this study had values greater than these cut-off points, as presented in Table 2. The construct “intention toward buying online,” was consistent with a Cronbach’s Alpha = 0.828 and CR = 0.880 (and adequate AVE = 0.597). As shown in Table 2, hedonic value had similar psychometric properties. Two constructs, attitude to online shopping (α = 0.574) and subjective norms (α = 0.586), originally fell below the recommended threshold of 0.70. Subjective norms item SN3 was associated with a poor factor loading (0.223) and non-significant t-value (*p* = 0.084), and so was deleted. After deleting SN3, the measurement model was re-estimated, and the reliability and validity of the subjective norms construct were successfully resolved. However, attitude” and perceived behavioral control still had both indicators showing Cronbach’s Alpha scores below an appropriate cut-off. Consequently, these constructs did not meet the cut-off recommendations. It is important to recognize that Cronbach’s Alpha is sensitive to the number of items in a construct and often underestimates reliability with few observed indicators. In these situations, an also better measure is CR and AVE showed accurate measures of a construct’s quality, and both criteria for these constructs were above appropriate cut-offs. Also, prior methodological literature describes that for new or exploratory research, Cronbach’ alpha of 0.50 is acceptable for early stage research, given the theoretical rationale and other reliability representations of the construct are satisfactory [119]. In review, while attitude and perceived behavioral control have slightly low alpha coefficients, the CR, AVE, and rho_A values suggest an acceptable degree of internal consistency and convergent validity to be included in the model. Additionally, as it was mentioned, perceived behavioral control (PBC) had a Cronbach’s Alpha of 0.612, which is comparable to previous reporting of PBC—while below its ideal range (0.70), still met minimum reliabilities with support of its CR and AVE values. Moreover, the rho_A values, a more precise measure of construct reliability in PLS-SEM, were consistently in acceptable range or slightly below in instances of less indicators. Thus, while minor reliability concerns remain for attitude and perceived behavioral control, the theoretical and methodological justifications outlined above support their retention, and the measurement model adjustments effectively resolved the issues with subjective norms. Taken in totality, the findings provide evidence of the internal consistency and convergent validity of the measurement model. In conclusion, despite marginally being below ideal thresholds for a few indicators, the measurement model demonstrates adequate psychometric soundness enabling the researchers to proceed confidently to the structural model analysis.

Table 3 provides the results of the measurement model, with standardized factor loadings and t-values for all indicators measuring the latent constructs in this model. Overall, and for the majority of the indicators, the factor loadings exceeded a factor loading threshold of 0.7 as suggested by Hair et al. [116]. Thus, indicating that the indicators are good explanations for the respective latent variables. For example, all of the perceived behavioral control (PBC), hedonic value (HV), utilitarian value (UV), attitude toward online grocery shopping (AGF), and purchase intention indicators had acceptable, statistically significant loadings (*p* < 0.001). In particular, the highest loading in the model is related to SN1 (0.924) and SN2 (0.890) representing the concept of subjective norms. The only discrepancy was found with SN3 (in initial running of the model) which loaded significantly lower at 0.223 with an associated t-value of 1.730 and was below the generally acceptable threshold (loading > 0.7; t > ±1.96). Therefore, it was eliminated from the model and eliminating it form the analysis improved the model reliability and validity criteria significantly. The second borderline example was PBC3 which had a factor loading of 0.665. Although this is less than the ideal cutoff of 0.7 it is above 0.60 which is acceptable, provided there is theoretical justification. Therefore, we kept this item in the analysis. In general, the significant and high factor loadings, coupled with significant t-values, confirm that the measurement model has good convergent validity and adequate reliability of indicators. These results provide evidence that the items were appropriate for use in the subsequent structural model test.

Discriminant validity is an essential characteristic of measurement models, because it indicates that each latent construct in a theoretical framework is also empirically distinct. Discriminant validity represents whether a construct is measuring a unique concept that is not being captured by other constructs. This study used the Fornell–Larcker criterion and HTMT to evaluate discriminant validity, which is a common, valid and established measure in structural equation modeling. The Fornell–Larcker criterion states that the square root of the Average Variance Extracted (AVE) for each construct should be greater than the correlation coefficients representing the relation between that construct and all other constructs in the model. Table 4 provides evidence of discriminant validity, which is when the diagonal elements of the matrix (which are the square roots of Average Variance Extracted) are higher than the corresponding off-diagonal inter-construct correlations (in their respective rows & columns); for example, the square root of AVE for the construct “hedonic value” is 0. 799, which is greater than the constructs “utilitarian value” (0.435), “subjective norms” (0.473), or “intention towards buying online” (0.593). Similarly, “perceived behavioral control in online shopping” had a square root AVE of 0.749, higher than its correlation with “attitude towards online shopping” (0.187), and “utilitarian value” (0.385). As found with each construct, “intention towards buying online” demonstrated a strong squared root AVE value of 0.772, higher than its strongest correlation (attitude—0.619).

The HTMT results confirm that the constructs exhibit acceptable discriminant validity across constructs, as all values are under the upper limit of the threshold of 0.90. The strongest relationship is between attitude towards online shopping and intention towards buying online (0.882), followed by intention towards buying online and hedonic value (0.772). Moderate correlations are shown between hedonic value and perceived behavioral control (0.753), and intention towards buying online with perceived behavioral control (0.718). The two weakest associations are attitude and perceived behavioral control (0.306) and subjective norms (0.344). The results confirm that all constructs developed in the model were well-separated and conceptually distinct from one another. Therefore, using the guidelines proposed by Fornell and Larcker, it was shown that this study’s measurement model achieves discriminant validity, which continues to enhance the overall construct validity of research instrument, and confidence when utilizing statistical tests that examine aspects of the structural model.

**Figure 2:**
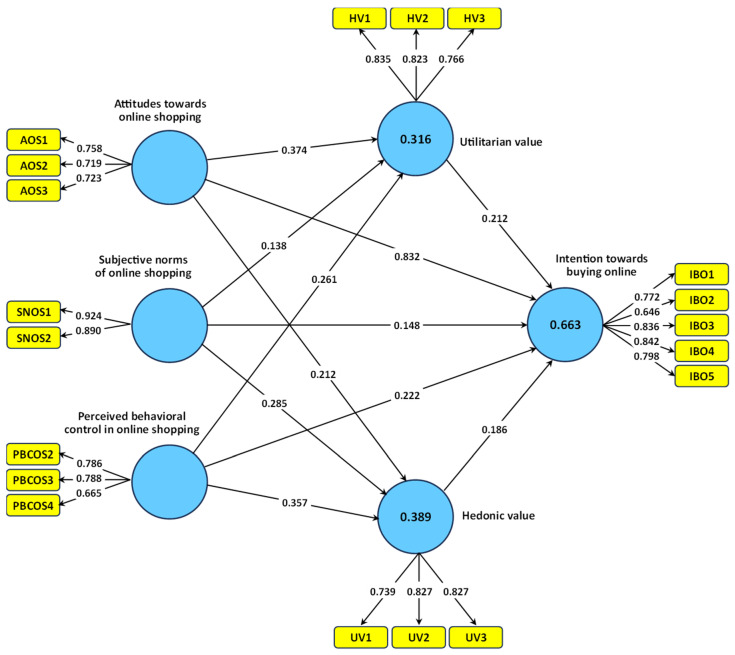
The PLS based SEM model with standardized path coefficients.

The findings in Table 5 and Figure 2 provide strong support for the structural model, especially the direct effects of core psychological constructs on consumers’ intention to buy local food products using online platforms. In terms of direct effects, attitude towards online shopping was the most powerful predictor (β = 0.382, T = 8.846, *p* < 0.001), reflecting the centrality of the favorable perceptions when forming behavioral intentions. This finding is consistent with TPB, where attitudes serve as the cognitive basis of intention [77]. Similarly, hedonic value (β = 0.186, T = 3.592) and utilitarian value (β = 0.212, T = 4.804) both had significant and substantial effects, suggesting that both emotional enjoyment and practical gain from an online shopping experience are also valuable motivational premises to consumer participation in this context. Perceived behavioral control (β = 0.222, T = 5.214) and subjective norms (β = 0.148, T = 3.255) also had significant effects—suggesting that consumers’ perceived ability to perform an online transaction and perceptions of social pressures from important others contributed meaningful towards their intention to purchase nomadic local food, using digital means.

In addition to these direct effects, the research also revealed that hedonic and utilitarian values also mediate how upstream psychological drivers (attitudes, social norms and perceived control) are transmitted into behavioral intention (Table 5). All six mediating effects in the model were statistically significant, indicating that motivational values act as essential cognitive-emotional conduits through which consumers interpret and respond to online shopping influences. The indirect effect of attitude through hedonic value (β = 0.039, T = 2.942) and through utilitarian value (β = 0.079, T = 3.644) reveal that attitudes not only stimulate an evaluation in terms of enjoyment, but also in relation to task completion with respect to online shopping that then lead to intention to buy. The same multi-faceted indirect influences of perceived behavioral control and subjective norms led to intention through hedonic and utilitarian values, suggesting that for individuals feeling they could do it, or were socially sanctioned to do it, led to increased enjoyment and usefulness in the process of online shopping. The significance and non-significance of these indirect effects were assessed using a basic bootstrapping procedure with 500 resamples, applying the Bias-Corrected and Accelerated (BCa) method at a 0.05 significance level. This approach provides more reliable confidence intervals for mediation effects and strengthens the robustness of the indirect effect testing. For the first time, the findings provide evidence that motivational values are not just outcomes of cognition, but also can serve and be understood as mediators in behavioral processes. Accordingly, the model presents a thinkers “layered” psychological approach to modeling human behavior that is increasingly integrated within instrumental, affective, and normative psychological frameworks to understand consumer behavior in culturally significant and emerging digital food markets.

Figure 2 provides the R Square (R^2^) values for the three endogenous constructs of hedonic value, utilitarian value, and intention towards buying online as an indicator of explained variance, or in other words how much the variation in each of the dependent constructs can be explained by the predictor variables in the structural model. R^2^ is a measure of how well the linear regression equation predicts the changes in the dependent variable. Statisticians categorized R^2^ values of 0.75, 0.50, and 0.25 as substantial, moderate, and weak explanatory power, respectively. Within that classification, the R^2^ value for intention towards buying online of 0.663 is moderate to strong which provides confidence that approximately 66.3% of the variation in consumers’ intention towards buying local food online was explained by the exogenous variables in the model including attitude, subjective norms, PBC, hedonic value, and utilitarian value. For utilitarian value, R^2^ is 0.316, whilst for Hedonic Value R^2^ is 0.389. Both should indicate moderate predictive utility. These values indicate that the hypothesized antecedents utilized (attitude, PBC and subjective norms) account for approximately 31.6% of the variance in utilitarian value and approximately 38.9% in hedonic value. While these figures do not attain a substantial increase for sufficient thresholds, they should still be seen as sufficient in behavioral and marketing research contexts [116]. Additionally, the relatively close value of the R^2^ and the Adjusted R^2^ across all three constructs provided an indication that the model does not have a problem fit to the complexity of the model or with regard to overfitting. Ultimately, the data indicate that this model is statistically adequate and has a degree of explanatory utility in a practical sense, in predicting the online purchase intention towards local food products.

In Figure 2, the model is divided into two sections known as the measurement model and structural model. The outer arrows (from the latent constructs to the indicators) of the model depict the measurement model, while the inner arrows (from the latent constructs to the latent constructs) differentiate the structural model. The arrows on the measurement model represent the measurement of each latent variable (i.e., attitude, subjective norms, perceived behavioral control, etc.) with its observed items (indicators). The measurements (in the measurement model) are the factor loadings depicted on each arrow. The factor loading indicates the strength or reliability based on one indicator and its underlying latent construct. The arrows on the structural model, indicating the hypothesized relationships among the study variables, represent where the relationships being tested fall into the structural model. The values shown on the structural model arrows are the path coefficients indicating both the strength and direction of the effect of one latent construct on another. Further to add insight into the model being examined are the values shown inside some of the construct boxes, which are the R^2^ values. The R^2^ values depict the amount of variance explained in the dependent variable by the independent variables that predict it. Thus, if the R^2^ value is higher comparative to another independent variable, then that independent variable represents a higher level of variance in comparison to the other dependent variable construct meaning that this independent variable has a stronger contribution to the model.

In order to evaluate the possible effect of Common Method Bias (CMB) in the model (which includes attitude, subjective norms, perceived behavioral control, utilitarian value, hedonic value and their direct effects on intention to purchase online) a marker variable technique was employed. A marker variable is a theoretically unrelated variable added into the model; in turn this is assessed to identify any significant parameters which may have changed, as a result of method variance. As shown in Table 6, and with respect to a threshold of 0.05 to identify CMB, the comparison of the marker variable against the original model showed limited impact in terms of changes in the path coefficients, as highlighted previously. The effect of attitude towards online shopping on intention decreased marginally from 0.382 down to 0.364 (Δ = 0.018), and the effect of subjective norms decreased from 0.149 downward to 0.132 (Δ = 0.016). The coefficient for perceived behavioral control decreased from 0.222 to 0.189 (Δ = 0.033), Utilitarian value evidenced diminishing returns from 0.212 to 0.196 (Δ = 0.016), and hedonic value evidenced similar declines from 0.186 to 0.174 (Δ = 0.012). As all differences observed are small differences (Δ < 0.05) and none of these impacts the statistical significance of the paths, this study does not contend that CMB has severely impacted findings. In addition to this, acquiescence bias (the propensity of respondents to agree with items with any content) was minimized through the use of balanced scale construction to include positively and negatively worded items. This method assisted in ensuring that the respondents were not able to agree uniformly across all statements, minimizing the possibility that the results were inflated by a response style rather than a meaningful assessment. The results of the structural model are valid and reliable.

Table 7 summarizes the effect sizes (f^2^) of the variables that positively influence intention to purchase online. Based on Cohen’s (1988) benchmarks, attitude towards online shopping is the most impactful variable, producing a medium-to-large effect size (f^2^ = 0.2198), recognizing it as the most influential predictor of online purchase intention. The small-to-moderate effect sizes of perceived behavioral control (f^2^ = 0.0712) and utilitarian value (f^2^ = 0.0628) indicate that consumers’ perceived ability to shop online and the utilitarian aspects of online shopping also add meaningfully, but to a lesser degree, to intention. Hedonic value (f^2^ = 0.0392) and subjective norms (f^2^ = 0.0371) provide small effect sizes, indicating enjoyment and social influence only slightly affect intention. The results demonstrate that consumers are affected by a number of factors influencing online purchase intention, yet, the attitude involving online shopping remains the dominating influence.

## 5. Discussion and Implications

The results of this study show the essential role of consumers’ attitudes towards online shopping as the main predictor of their intention to purchase nomadic and local food products on e-commerce sites such as Ashayershop and others. This is congruent with the overarching premise of the Theory of Planned Behavior [77], which is backed by previous empirical evidence in the e-commerce literature (e.g., [84,85]) that a positive attitude is a strong predictor of purchase intention. If the consumer finds shopping on sites such as Ashayershop efficient, enjoyable and trustworthy, they are more likely to accept such services, especially with traditional nomadic food products. This highlights the necessity for good user experiences and emotional trust in their delivery. Thus, business in this sector should develop user interfaces that are user-friendly, visually appealing and culturally connected to their customers, as well as emotionally appealing through design to customers’ values. Marketing communications that marketing reliance that draws on tradition and the innovation of online shopping channels offers an added incentive to further promoting consumers’ positive attitudes towards online purchase channels.

Hedonic value is also one of the significant factors that contributed to purchase intention, suggesting that consumers’ affective and experiential enjoyment of using Ashayershop has an impact in this context. This corroborates the operationalization found in previous research of hedonic motivations such as an emphasis on fun or pleasure (e.g., [88]), where motives like entertainment and pleasure are stronger for non-utilitarian shopping. As nomadic food products often have emotional and socio-cultural connections, the hedonic aspect is especially important. Thus, this type of setting should promote rich storytelling, visually engaging or immersive elements (e.g., videos of food being prepared), and culturally relevant stories to deepen the consumers’ emotional shopping experience. Digital aspects of engagement such as gamification, and community-based comments and reviews to encourage hedonic perceptions of users to enhance purchase intentions would be ideal. Also included was utilitarian value, meaning that the usefulness of online shopping such as convenience, efficiency, and cost effectiveness was found to play a role here, as well. While utilitarian value has been severely underrepresented in much of the mainstream e-commerce literature (e.g., [88]), the next round of research demonstrates that even in markets where products are culturally significant, use-value is still a salient consideration. Thus, once validated, sites like the Ashayershop need to showcase not only the distinctiveness and heritage of nomadic foods, they also need to stress their logistical advantages, such as reliable payment methods, flexible delivery and pricing. A particular consideration when serving semi-urban and rural communities is that in the case of limitations to the infrastructure that would not allow for high reliability in delivery, and high levels of transparency for transactions, still allows the Ashayershop to offer incremental increases in perceived use-value.

Perceived behavioral control was identified as a key predictor of purchase intention, meaning that consumers’ confidence in their ability to, take transactions on these platforms, is really important. This finding is consistent with Ngo-Thi-Ngoc et al. [83] and Chilón-Troncos et al. [84] and inconsistent with Dangelico et al. [120] in the past research. The results of this section suggest a need to minimize perceived barriers in the customer journey. When there is a lack of digital literacy or access, potential users may be put off by using these platforms, especially for niche products like nomadic foods. To altogether reduce any uncertainty in potential users, businesses should have simplified interfaces, perhaps instructional videos in local languages and considerations for offline support services. Businesses may also work with some rural cooperatives and local ambassadors to make it easier for new customers to engage with the platforms.

The impact of subjective norms implies that social validation, and social influence from significant others, is crucial to consumer decisions to purchase local foods online. This aligns with the findings of Ngo-Thi-Ngoc et al. [83] and Chilón-Troncos et al. [84] that social norms have particularly strong influence over intention adoption of consumers. In traditional cultures, there is often community or family involvement in the purchasing process, so endorsement from family, friends, or community leaders is paramount. Therefore, online spaces should consider suggestions, testimonials, peer endorsement, as well as collaborations with valued local ambassadors and influencers. Community education campaigns, alongside endorsement from cultural communities, and chefs may build social legitimacy for the practice of purchasing local foods and increase rates of adoption.

Critically, the research demonstrates that hedonic and utilitarian values are both mediators in the relationship between psychological antecedents (attitude, perceived control, and subjective norms) and purchase intention. This tabled mediation shows that even when making future purchasing intentions, consumers act using cognitive or social factors, but cognitive-affective valuations on the online shopping experience. Although prior studies have already suggested that hedonic and utilitarian values can add depth to the TPB framework (e.g., [88]), our results ground this extension in the cultural and heritage food sector. In this space, hedonic and utilitarian values do not simply serve as additional predictors, but act as mediating mechanisms of converting attitudes, subjective norms, and perceived behavioral control into behavioral intention. It should also be further emphasized that there may be some conceptual overlap with utilitarian value and the idea of “positive attitude,” due to utilitarian value’s functional nature; however, utilitarian value is treated as a separate motivational construct in this study. That is, utilitarian value acts as a mediator to direct the effect of broader patterns of attitudinal, normative and control beliefs into purchase intention. This adds further consistency with terminology and conceptualization in our study. Furthermore, our research sheds light on some boundary conditions of the TPB extension: hedonic and utilitarian values are more consequential within contexts involving products that are culturally or emotionally embedded, or are consumed in a social context. When compared to other frameworks like the Technology Acceptance Model (TAM) or Value-Belief-Norm (VBN) theory, the TPB with motivational values can provide a more thorough representation of both cognitive and affective factors associated with intention, thereby interfacing social, emotional, and functional influences. This provides additional insights into how motivational values work in markets where cultural identity intersects with digital commerce. These findings also broaden the Theory of Planned Behavior by highlighting motivational values as cognitive-affective conduits, with the clear message that the platforms need to be able to transform changes in beliefs into enriching emotional and functional experiences. An integrative approach across education, social appeal, emotional appeal, and functional design will be important for increasing user engagement in a more holistic manner.

An interesting implication of this mediation is the complicated connection between subjective norms and hedonic value. Whereas the direct effect of social norms on intention was revealed to be moderate, the indirect effects of social norms via hedonic and utilitarian values were substantial. In other words, social norms can increase people’s emotional and utilitarian perceptions of online shopping. Ultimately, if marketers can show that digital shopping is socially acceptable and emotionally rewarding, for example, by depicting families eating meals from these platforms in their advertising, they can enhance both aspects of shopping and influence consumer intention.

In summary, these findings contribute to theoretical and practical understanding. In theory, they challenge TPB by demonstrating how hedonic and utilitarian values are functioning as mediators when specific cultural and social boundary conditions are taken into account, and they provide a more integrative alternative to frameworks such as TAM or VBN. In practice, they provide a framework that is actionable and will inform differentiated strategies with applied implementation guidelines and KPIs for different consumer segments. Future studies could examine these relationships in additional culturally grounded contexts and further investigate how digital transformation interacts with heritage consumption in emerging markets.

## 6. Conclusions, Limitations, Future Direction

### 6.1. Main Conclusions

This study highlights the central role that consumers’ attitudes, perceived behavioral control, and subjective norms have, in forming their intentions to purchase nomadic and local food products online through sites like Ashayershop. The evidence further establishes that both hedonic and utilitarian values, as positive attitudes, play a noteworthy role in influencing consumers’ intention to purchase. Hedonic aspects are emotionally or culturally derived attributes to being able to engage in the online robotic consumer process, while utilitarian aspects like convenience and dependability help consumers address their utilitarian requirements when purchasing these items. The mediating role of the motivational values also adds some explanation to these consumer actions through the lens of the Theory of Planned Behavior, identifying the role of cognitive and affective characteristics. Ultimately, these findings have implications for e-commence businesses, local food producers, and designers of policy that aim to assist the digitization of traditional food markets to respond to consumers’ changing needs in a culturally laden context.

### 6.2. Study Limitations

This study has added to the body of knowledge regarding types of consumers associated with online food shopping, but certain limitations must also be addressed. First, the sample used in this study was limited to users ordering from companies like Ashayershop which may limit the generalizability to online food platforms in other places or providers of different types of food, which includes agri-food and local food. So, the findings are to be read context-specifically and not to assume [they represent] all of consumers or all of online food platforms making the findings somewhat a apocryphal. Second, we relied on self-reported data which may present social-desirability bias in our answers or may not fully capture actual purchasing behavior, so scope for strong causal inferences is limited. This is compounded by focusing only on nomadic and local food products without exploring consumer behavior on a wider range of culturally wise products. Finally, as digital commerce continues to evolve quickly in light of technologies that innovate and promote new digital business models, the findings presented here may not reflect behaviors modeled sharply by emerging technologies. Therefore, keeping abreast of trends, market activity, and research developments in this area of study will be important. Moreover, examining comparisons of active online users with a control group and/or comparisons based on prior use of the online portal as a segmentation criterion would help to provide greater understanding of consumer behavior. However, these analyses fall outside the current manuscript length restrictions, and have been noted as limitations here instead. It should be acknowledged that detailed individual-level demographic data (e.g., age, gender, lifestyle) were not systematically collected, limiting the ability to analyze individual variation in responses. The study presents aggregated demographic data for the sample, which cannot be directly linked to individual cases. Furthermore, filter and segmenting questions were not included, which restricts the multidimensionality of the analysis. These factors represent limitations in understanding the full diversity of consumer behavior and will be addressed more comprehensively in future studies.

### 6.3. Future Research Directions

Future research opportunities exist that could address the limitations of this study, as well as building a deeper understanding of online food consumer behavior. Increasing the sample size from different geographical regions, cultural backgrounds, and demographic factors would potentially improve generalizability as well. Researchers could also make use of longitudinal or behavioral data to better understand actual purchasing behavior rather than depending solely on self-reported buying behavior over longer periods of time, as well as providing stronger constraining causal interpretations of consumer behavior patterns by identifying potential causal effects. On the other hand, the nature of collecting behavioral or longitudinal data in rural and nomadic contexts poses some practical challenges, such as lacking internet, seasonal mobility, and varying degrees of digital literacy. Researchers can find solutions by using mobile survey software that can operate offline, and then throughout the study, researchers will sync the surveys when there is connectivity; partnering with a local community intermediary or platform manager to monitor anonymized purchasing records longitudinally; or using mixed methods by coupling short-term digital tracking (in a responsible way) with qualitative in-depth interviews. Researchers can also frame a calendar timeline to engage people and to promote community engagement for continued research participation over these longitudinal periods. It could also be interesting to explore other product categories beyond food, while including both traditional and more contemporary categories of products to see how consumer engagement may differ, particularly with regard to types of culturally important offerings. Finally, researchers could investigate the impacts of other Iranian digital technologies such as sarvabab, Parsiano, Keshmoon, and Sava platforms. Understanding how these innovative technologies and platforms are impacting trust, engagement, and purchase intention could also help inform marketers and developers of rural and nomadic digital food platforms. These more analytically related approaches could generate a richer understanding of consumers’ continuously developing and changing behaviors in the online food marketplace, and theoretically inform enthusiasts and practitioners on engagement, intention to purchase, trust or selection on the use of platforms for reading food products. More specifically, future studies could include comparisons with a control group or categorize users based on prior full portal usage frequencies. This would be a meaningful way of addressing the limitations noted, as well as increasing understanding around online consumer behavior.

## Figures and Tables

**Table 1:** Items used to measure the constructs.

Var.	No.	Items	Source
Perceived behavioral control	Perceived behavioral control		[59]
	1	Buying local food products from the Ashayershop platform is easy for me.	
	2	I have sufficient control to easily make purchases from Ashayershop.	
	3	If I wanted to, I could easily buy from the Ashayershop platform.	
Intention	Intention		[114,115]
	1	I am willing to buy local food products from the Ashayershop platform again	
	2	I intend to use this platform to purchase food products in the future.	
	3	I recommend buying local food products from the Ashayershop to others	
	4	I actively look for opportunities to purchase from the Ashayershop platform	
	5	I am likely to make more purchases from Ashayershop in the future	
Subjective norms	Subjective norms		[69]
	1	Important people to me (family, friends) believe that I should purchase food products from the Ashayershop platform.	
	2	There is social pressure for me to buy local food products online from Ashayershop.	
	3	I feel that others expect me to use the Ashayershop platform for purchasing food products.	
Attitude	Attitude		[69]
	1	Purchasing local food products from the Ashayershop platform is a good choice.	
	2	Using the Ashayershop platform gives me a sense of satisfaction.	
	3	Overall, buying local food products online from the Ashayershop is a positive and valuable experience for me.	
Utilitarian Value	Utilitarian Value		[114,115]
	1	Buying local food from the Ashayershop and its services is convenient and fast	
	2	Purchasing food through the Ashayershop platform is highly practical and economical	
	3	The Ashayershop platform offers a diverse menu of healthy and tasty local food products.	
Hedonic Value	Hedonic Value		[114]
	1	Buying local food from the Ashayershop platform is a pleasant, enjoyable, and exciting experience for me	
	2	Using the Ashayershop platform gives me a good feeling	
	3	Browsing and shopping for local food through the Ashayershop is entertaining for me	

**Table 2:** Measurement items and indicators of model fit.

Construct	Cronbach’s Alpha	Composite Reliability	Average Variance Extracted (AVE)	rho_A
Attitude towards online shopping	0.574	0.778	0.538	0.574
Hedonic Value	0.717	0.841	0.638	0.728
Intention towards buying online	0.828	0.880	0.597	0.842
Perceived behavioral control in online shopping	0.612	0.792	0.560	0.629
Subjective norms of online shopping	0.785	0.902	0.822	0.802
Utilitarian Value	0.735	0.850	0.654	0.734

Acceptable values for the reported indices: Alpha > 0.7; *p* < 0.01; CR > 0.7; and AVE > 0.5.

**Table 3:** Measurement items, Loading factors and T-value of the model.

Factors	Indicators	Loading Factor	T-Value	Significant	Result
PBC	PBC1	0.786	27.826	0.001	Accepted
	PBC2	0.788	19.151	0.001	Accepted
	PBC3	0.665	11.302	0.001	Accepted
HV	HV1	0.835	13.152	0.001	Accepted
	HV2	0.823	27.125	0.001	Accepted
	HV3	0.766	30.594	0.001	Accepted
SN	SN1	0.924	67.859	0.001	Accepted
	SN2	0.890	46.126	0.001	Accepted
UV	UV1	0.739	31.076	0.001	Accepted
	UV2	0.827	35.047	0.001	Accepted
	UV3	0.827	21.753	0.001	Accepted
AOS	AOS1	0.758	24.213	0.001	Accepted
	AOS2	0.719	18.675	0.001	Accepted
	AOS 3	0.723	17.898	0.001	Accepted
Intention	Intention1	0.722	18.943	0.001	Accepted
	Intention2	0.646	10.156	0.001	Accepted
	Intention3	0.836	35.696	0.001	Accepted
	Intention4	0.842	31.658	0.001	Accepted
	Intention5	0.798	24.141	0.001	Accepted

Acceptable values for the reported indices: all loadings > 0.7; *p* < 0.01; CR > 0.7; and AVE > 0.5; T value > ±1.9.

**Table 4:** Assessment of the discriminant validity.

Fornell–Larcker Criterion						
	1	2	3	4	5	6
Attitude towards online shopping	0.734	--	--	--	--	--
Hedonic Value	0.345	0.799	--	--	--	--
Intention towards buying online	0.619	0.593	0.772	--	--	--
Perceived Behavioral Control in online shopping	0.187	0.507	0.527	0.749	--	--
Subjective Norms of online shopping	0.234	0.473	0.481	0.387	0.907	--
Utilitarian Value	0.455	0.435	0.601	0.385	0.327	0.809
Heterotrait-Monotrait Ratio (HTMT)						
Attitude towards online shopping	--	--	--	--	--	--
Hedonic Value	0.540	--	--	--	--	--
Intention towards buying online	0.882	0.772	--	--	--	--
Perceived behavioral contol in online shopping	0.306	0.753	0.718	--	--	--
Subjective norms of online shopping	0.344	0.618	0.593	0.531	--	--
Utilitarian Value	0.693	0.596	0.753	0.569	0.419	

**Table 5:** Estimated effects on intention.

Hypothesis	Direct Effects		Indirect Effects		*p* Value	Result
**Direct effects on Intention**	**T**	**Beta**	**T**	**Beta**		
Attitude towards online shopping → Intention towards buying online	8.846	0.382	--	--	0.000	Confirmed
Subjective Norms of online shopping → Intention towards buying online	3.255	0.148	--	--	0.001	Confirmed
Perceived Behavioral Control in online shopping → Intention towards buying online	5.214	0.222	--	--	0.000	Confirmed
Utilitarian Value → Intention towards buying online	4.804	0.212	--	--	0.000	Confirmed
Hedonic Value → Intention towards buying online	3.592	0.186	--	--	0.000	Confirmed
**Meditated indirect hypotheses**						
Attitude towards online shopping → Hedonic Value → Intention towards buying online	--	--	2.942	0.039	0.003	Confirmed
Perceived Behavioral Control in online shopping → Hedonic Value → Intention towards buying online	--	--	3.068	0.067	0.002	Confirmed
Subjective Norms of online shopping → Hedonic Value → Intention towards buying online	--	--	2.511	0.053	0.012	Confirmed
Attitude towards online shopping → Utilitarian Value → Intention towards buying online	--	--	3.644	0.079	0.000	Confirmed
Perceived Behavioral Control in online shopping → Utilitarian Value → Intention towards buying online	--	--	3.324	0.056	0.001	Confirmed
Subjective Norms of online shopping → Utilitarian Value → Intention towards buying online	--	--	2.410	0.029	0.016	Confirmed

**Table 6:** Common method Bias (CMB) assessment though including a marker variable.

Direct Effects on Intention	Beta (Original Model)	Beta (Marker Variable Included)	Difference of Beta
Attitude towards online shopping → Intention towards buying online	0.382	0.364	0.018
Subjective Norms of online shopping → Intention towards buying online	0.148	0.132	0.016
Perceived Behavioral Control in online shopping → Intention towards buying online	0.222	0.189	0.033
Utilitarian Value → Intention towards buying online	0.212	0.196	0.016
Hedonic Value → Intention towards buying online	0.186	0.174	0.012

**Table 7:** Effect sizes of the variable.

Direct Effects on Intention	f^2^ (Variable Included)	f^2^ (Variable Excluded)	Effect Size
Utilitarian Value	0.663	0.634	0.0628
Hedonic Value		0.645	0.0392
Attitude towards online shopping		0.555	0.2198
Subjective norms		0.646	0.0371
Perceived behavioral control		0.630	0.0712

## Data Availability

The original contributions presented in the study are included in the article, further inquiries can be directed to the corresponding author.
